# Paper-based PCR method development, validation and application for microbial detection

**DOI:** 10.1186/s43141-020-00110-1

**Published:** 2021-03-01

**Authors:** Amruta Patil-Joshi, B. E. Rangaswamy, Anjali Apte-Deshpande

**Affiliations:** 1 Department of Biotechnology, BIET, Davangere, Karnataka 577004 India; 2 Central Dogma Pvt Ltd., A4, Gulmohar Residency, Baner Road, Baner, Pune, Maharashtra 411045 India

**Keywords:** Coliforms, Milk sample, PCR, Rapid test, Whatman paper

## Abstract

**Background:**

The analysis of the quality of food is important to protect humans from food-borne or food-based illnesses caused by pathogens, such as bacteria, fungi, viruses, and protozoa. Rapid identification of these pathogens is critical to ensure food safety. Various detection and identification strategies exist; however, they are laborious and time consuming and hence the detection takes longer time. The aim of this study was to develop the specific and fast method for the detection of contaminants in milk.

**Results:**

In this study, we have developed a simple paper-based PCR method with minimum sample preparation process. The 16S rDNA universal primers were used for the detection of bacterial contaminants. LacZ primers were used for coliform detection which causes serious illness and hence their detection is crucial. ITS region primers were used for fungal detection. The most unique thing about this study is use of Whatman paper no. 1 as sample carrier material. We developed and validated the paper-based PCR method and used it for the detection of microbes and coliforms using milk as a representative sample.

**Conclusion:**

We evaluated this method for its suitability in the detection of contaminant microbes using different milk samples. The paper-based method could successfully detect contaminants in the milk samples and the results were comparable to the traditional microbial detection method. The traditional microbiological method takes at least 18–20 h for detecting the presence of microbes in any sample but the developed paper-based PCR method can confirm the microbial presence in 2–3 h. This is very promising especially in the testing where sample sterility is crucial.

## Background

The foodborne pathogens can enter through contaminated and undercooked food. This can lead to serious illnesses in short duration and sometimes it can be fatal. Hence, it is important to detect the presence of pathogens in the food and water before it enters the body to cause a serious problem [1]. Traditionally, identification of the most microbial pathogens in food involves enrichment of the cultures, cultivation on selective media, and ultimately a series of biochemical tests to identify the organisms. Such standard microbiological techniques are slow, laborious, and often require several days, even weeks, to be performed. Enrichment protocols also may fail to detect strains of bacteria present in the food if they are at low levels. A closer observation at methods available for the detection of organisms like total coliform and *E. coli* reveal that, majority of these methods have incubation at 37 °C for 24 h to obtain the results. Hence, researchers are exploring various fast detection techniques. Recently, scientists have worked on faster, more specific, less expensive, and highly sensitive methods to detect and identify microbial pathogens in the food samples. A study by Batule et al. [2] has even focused on a paper-based rapid method for extraction of mitochondrial DNA from processed meat. Paper-based detection system has been used by scientists for many years. Filter paper was first used as a scientific tool in 1815 by the Swedish chemist Jons Berzelius. In the 1940s, Heatley described the use of filter paper for incorporating antimicrobial solutions in Oxford, giving rise to antibiotic susceptibility disc testing [3].

Several reports indicate polymerase chain reaction (PCR) as a promising new diagnostic method to detect food borne pathogens [1, 4–6]. Most of the paper-based biosensors use the antigen-antibody interactions to detect the target analytes of interest in water, soil, urine, blood, or saliva samples [7–15]. Qi et al. [16] has in fact developed a paper-based device for the detection of antigen using molecular imprinting without the need of antibody. Applications built on paper-based sensing technology are numerous ranging from testing of blood samples for infectious diseases, testing of grains in agriculture to testing of chemical contaminants in water and soil [12–15, 17–19]. Truong et al. [20] used paper-based scaffolds for culturing mammalian cells and they further used qPCR for to quantify the breast cancer cells in these scaffolds. Fobel et al. [21] developed a paper-based microfluidic device to detect presence/absence of bacteria using chromogenic substrates. The bacteria in water samples are pre-concentrated using antibody-coated immune-magnetic nanoparticles and then tested with the paper-based microfluidic device. Similarly, Lin et al. [22] recently showed in their study that a PUA cured paper-based device can potentially detect microbes present in some aggressive liquids. Molina et al. [23] used a different strategy for differential identification of bacteria with the aid of multiplex PCR. Oligonucleotide primers were designed to ensure the specificity for the detection of *E. coli* and total coliforms in single assay. These genetic methods provide results in lesser time with excellent specificity. However, these genetic methods require high-end, expensive instruments and/or sensors. Also, these methods require more time in sample preparation before testing.

The goal of this study was to optimize a direct paper-based PCR technique for the detection of bacteria, coliforms, and fungi from food samples without the DNA extraction and time-consuming sample preparation process. To establish the proof of concept for this technique, PCR-based detection method development and optimization was done using the 16S rDNA gene, β-galctosidase gene, and ITS gene. These are the most common genetic markers used to study bacteria, coliform, and fungi, respectively. The 16S rDNA gene consists of conserved and variable areas. The conserved regions are targeted by prevalent primers for the detection of the presence of microorganisms in a given pattern, while the variable regions are targeted for the identity of genus or species [24]. The current work is a proof of concept to establish direct amplification of 16S rDNA, β-galctosidase gene, and ITS gene from a sample carried on a Whatman paper to evaluate the presence of microbes without extraction of genomic DNA. Known positive-control and negative control were run with each PCR amplification.

## Methods

### Cultures used

*E. coli* (ATCC8739) *Staphylococcus aureus* (ATCC25923), *Salmonella typhi* (ATCC23564), *Klebsiella pneumoniae*(ATCC), *Psudomonas aeroginosa* (ATCC9027), and *Bacillus subtilis* (ATCC6633), *Candida albicans* (ATCC10231) were procured from NCCS, Pune.

### Media and reagents

PCR reaction components like Taq polymerase, buffer, and dNTPs were from Invitrogen. Whatman filter paper no. 1 was from GE Healthcare Life Sciences. One hundred base pair ladder was procured from Genie, and Luria–Bertani (LB) agar components, MacConkey agar (MA), and potato dextrose agar (PDA) were procured from Himedia.

### Method development

#### Bacterial genomic DNA extraction

Genomic DNAs (gDNA) of *E. coli*, *S. aureus*. and *C. albicans* were isolated using QIAamp DNA Mini Kit from Qiagen following the protocol recommended by the manufacturer. Additionally, an isolated colonies of the strains mentioned above were picked up from LB and PDA agar plates. Colonies were re-suspended in 25 μL of water in distinct tubes. These tubes were kept in a boiling water bath to lyse the cells. The boiled cell suspension was spun down and the supernatant containing the gDNA was used as a template. The gDNA extracted using both the above methods were used as a template in the PCR.

#### Primers used

Two sets of universal primers with the following sequences were synthesized (Sigma) for amplification of the 16S rDNA gene. Set 1: F1: 5′ACT CCT ACG GGA GGC AGC AGT 3′, R1: 5′TCA CCG GCC GTG TGT ACA AG-3′ with amplicon size of 1086 bp [17] Set 2: F2: 5′GTG TAG CGG TGA AAT GCG 3′, R2: 5′ACG GGC GGT GTG TAC AA3′ with amplicon size of 709 bp [25]. For fungal detection, ITS primers FP-5′TCC GTA GGT GAA CCT GCG G3′ and RP-5′TCC TCC GCT TAT TGA TAT GC 3′ [26] with amplicon size of 500 bp and for coliform detection, primers LacZ3F-5′′TTG AAA ATG GTC TGC TGC TG 3′′ and LacZ3R-5′′ TAT TGG CTT CAT CCA CCA CA 3′′ [23] with amplicon size of 234 bp were used. Primers based on the LacZ gene have been used for the detection of coliforms because conventional coliform monitoring methods are based on the expression product (beta galactosidase) of this gene [27, 28].

#### Annealing temperature optimization

To evaluate the optimal annealing temperature for all primer sets mentioned in the above section, a gradient PCR program was set up in thermal cycler with an annealing temperature range of 55–58 °C. The PCR program was set as, 94 °C for 7 min, followed by 35 cycles of denaturation at 94 °C for 30 s, annealing 55–58 °C for 1 min, extension at 72 °C for 30 s, followed by final extension at 72 °C for 7 min. As a model organism, a single colony of *E. coli*, as well as *S. aureus* and *Candida albicans* strain were suspended in 20 μL of sterile water separately. Colony suspension was boiled for 7 min at 100 °C; it was spun and clear supernatant was spotted on pre-sterilized Whatman filter paper no. 1, and the paper was dipped in the master mix having all PCR components. This tube was placed in thermal cycler and the PCR program was run as mentioned above. The annealing was carried out at 55, 57, and 58 °C along with optimal concentrations of PCR reagents as 25 μM of each dNTP, 0.25 μM each primer, 0.5 U Taq polymerase, and 1× buffer. The amplicon was visualized with the use of 2% agarose gel, because of LacZ amplicon size was 234 bp and ITS amplicon size was 500 bp. The annealing temperature range mentioned above is tried for amplification of all three genes.

### Spotting of culture/sample on Whatman no. 1 paper

**Fig. 1:**
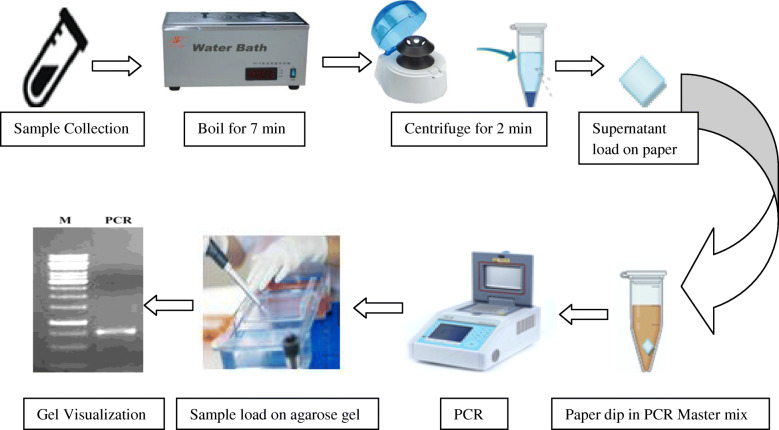
Schematic illustration of the developed Paper based PCR method

During the course of this study, Whatman paper no. 1 was used as a carrier source for the culture/sample. The paper was cut into desired size squares (1 × 1, 2 × 2, or 5 × 5 mm). These squares were wrapped in aluminum foil and were sterilized by steam sterilization using autoclave. If colony suspension is to be spotted on this paper, the colony suspension was prepared by dipping one colony in sterile water and the suspension was boiled for 7 min and then specific amount (2 uL/5 uL) of suspension was spotted on the sterilized paper square under aseptic condition. The paper was allowed to dry under aseptic condition and then was directly dipped in the PCR master mix. The PCR tube carrying this paper in the master mix was placed in the thermal cycle for further amplification. The experimental design is as depicted in Fig. 1.

When milk sample was spotted on the paper, same methodology mentioned above was followed, except the milk sample is boiled for 7 min instead of colony suspension. Colony suspension served as a representation of the sample during method development. When a specific sample like milk/water/juice needs to be tested for microbial load, it can be directly spotted on the paper without isolation/enrichment of bacteria from these sources.

### Robustness

### Bacterial culture volume and Whatman paper size optimization for paper-based PCR

As the paper-based PCR method for amplification of the 16S rDNA gene, β-gal, and ITS gene was established using Whatman filter paper no.1, it was important to optimize the lowest volume of bacterial culture that is sufficient to amplify the respective genes. Since the paper was directly dipped in 25 μL of PCR master mix in a PCR tube, there was a limitation of paper size to be used and hence the volume of culture accommodated on it. Hence 2, 3, and 5 μL of culture was spotted on the paper with dimensions, 1 mm × 1 mm, 3 mm × 3 mm, and 5 mm × 5 mm, respectively. *E. coli*, *S. aureus*, and *C. albicans* colony suspensions were prepared in sterile water. This suspension was directly spotted on three independent pre-sterilized small strips of Whatman filter paper no.1 of sizes mentioned earlier. The strips were allowed to air dry in LAFU and were directly dipped in 25 μL of PCR master mix and the amplification and detection were carried out. Standard colony PCR of the same culture using direct culture supernatant as a source of genomic DNA was also carried out along with this reaction as a positive control.

The16S rDNA gene is a housekeeping gene for all the bacteria, both for Gram-positive and Gram-negative. To ensure the robustness of the paper-based PCR to detect this gene, various Gram-positive and Gram-negative bacterial colony suspensions were prepared, and were spotted on pre-sterilized Whatman filter paper no. 1 as mentioned earlier. The bacteria used for this study were *Staphylococcus aureus*, *Klebsiella pneumoniae*, *Salmonella typhi*, and *Bacillus subtilis* sp. Each of these cultures was streaked on a sterile Luria–Bertani agar plate to achieve a single isolated colony. A single colony of each of these cultures was picked up and suspended in 20 μL of sterile water. Two microliters of each suspension was spotted on pre-sterilized independent Whatman filter paper no. 1 on 1 mm × 1 mm size strips and the strips were air-dried in LAFU. These strips were dipped in the master mix, and PCR condition was carried out as mentioned earlier in the case of annealing temperature optimization section, and the annealing temperature used for the amplification of all the genes was 57 °C. The experiment was carried out with F2/R2 primer set for 16S rDNA amplification.

#### Master mix volume variation

As the paper-based PCR method for amplification of the 16S rDNA gene, β-gal, and ITS gene was established using Whatman filter paper no.1, it was important to optimize the PCR master mix volume to check its effect on amplicon. Colony suspension was prepared in sterile water. These suspensions were directly spotted on pre-sterilized small strips of Whatman filter paper no.1 in 2 μL of volume. The strips were allowed to air dry in LAFU and were directly dipped in 25 μL and 50 μL of PCR master mix. The amplification of the gene and the detection of amplicon were carried out. Standard colony PCR of the same culture was also carried out along with this reaction as a positive control. Method robustness was also checked by inter-day and intra-day experimental set up as well as analyst variation (data not shown).

#### Specificity of primers for 16s rDNA, LacZ3, and ITS regions

Confirming the specificity of 16S rDNA primers for the detection of all bacteria by using established paper-based PCR method was important. The cultures used for this study were, *E. coli* and *Candida albicans* because *C. albicans* would not give amplification of 16S rDNA region as it is absent in the genome. Two microliters of each suspension was spotted on sterile 1 mm×1 mm size Whatman no.1 paper squares and was allowed to air dry in aseptic conditions. The paper was then added to the master mix, and the PCR was carried out as before. Specificity of LacZ3 primers for the detection of coliforms was evaluated using both coliform (*E. coli*.) and non-coliform (*S. typhi*.) cultures. Respective bacterial suspensions were spotted on the paper as mentioned earlier and the paper was added to the master mix. The PCR was carried out as mentioned above. Specificity of ITS primers for the detection of fungi was assessed by using cultures of *Candida albicans* and *E. coli.* because of *E. coli.* genome does not carry ITS region and hence should not give amplification with ITS primers. Two microliters of each suspension was spotted on sterile 1 mm × 1 mm size Whatman no.1 paper squares and was allowed to air dry in aseptic conditions. The paper was added to the master mix. The methodology of spotting cultures and PCR amplification was same as mentioned earlier. Post amplification, the amplified product was loaded on 2% agarose gel having ethidium bromide and the amplicon was visualized on UV transilluminator.

#### Evaluation of paper-based PCR method for the detection of contaminants in milk samples

The applicability of the developed paper-based PCR method for the detection of bacteria and coliforms was tested with different sources of milk. The milk sources used were cow milk from local dairy, buffalo milk from local dairy, pre-packed Amul milk, and pre-packed Gokul milk from Pune, Maharashtra.

In this study, we compared two methods viz. traditional microbiological method and developed paper-based PCR method (molecular biology-based method). In traditional microbiological method, we followed the process mentioned by Miles and Mishra [29] to detect the total bacterial contaminants and their count by the colony formation unit per mL (CFU/mL). Samples were serially diluted with 10-fold dilutions up to10^−6^ and were spotted on LB agar plates as mentioned in the article by Miles and Mishra [29]. The CFU/mL of each sample was calculated as per microbial method detection.

#### Detection of microbes in milk by paper-based PCR method

Twenty microliters of milk sample was transferred into sterile tube and boiled at 100 °C for 7 min. Contents were spun down and 2 μL supernatant of respective milk sample was spotted on 1 mm × 1 mm size of Whatman paper no. 1 under sterile condition. This paper was dried in LAFU. Then dried paper was dipped into PCR master mix tube. PCR was carried out with each selected milk source. Both 16S rDNA and LacZ3 primers were used in the PCR for the detection of bacteria and coliforms. A negative control reaction was also set up. Post amplification, the amplified products were loaded on 2% agarose gel having ethidium bromide and were visualized on a UV transilluminator

## Results

### Amplification of 16S rDNA, LacZ3, and ITS genes with selected primers using colony suspension

**Fig. 2:**
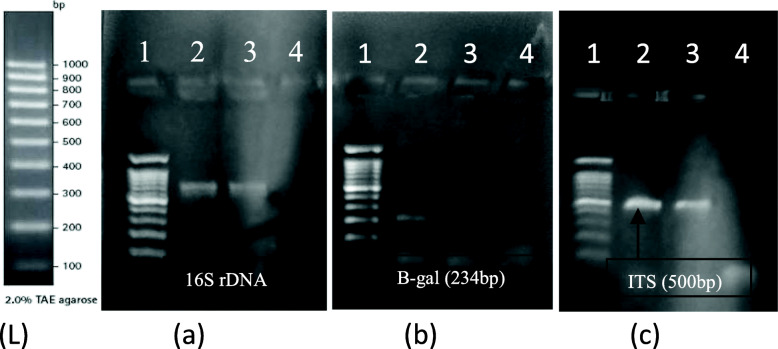
16s rDNA, LacZ3, and ITS region amplification using genomic DNA and colony as a template. (L)-100 bp Ladder. **a** 16s r-DNA: lane 1—100 bp ladder, lane 2—genomic DNA PCR, lane 3—colony PCR, lane 4—negative control. **b** β-galactocidase: lane 1—100 bp ladder, lane 2—genomic DNA PCR, lane 3—colony PCR, lane 4—negative control. **c** ITS: lane 1—100 bp ladder, lane 2—genomic DNA PCR, lane 3—colony PCR, lane 4—negative control

The purpose of the paper-based PCR method was mainly to detect bacterial presence directly from the sample without the requirement of bacterial genomic DNA extraction. It was therefore important to ensure if the selected primers can give desired amplicon from cell suspension and if it is of the same size and specificity when genomic DNA is used as template. As is evident in Fig. 2, both extracted genomic DNA and colony suspension were used as template in PCR for amplification all three genes and the amplicons were compared with 100 bp ladder. Colony suspension could give amplification for all three genes. This data indicated that amplification does not affect the type of template source.

### Primer annealing temperature optimization for amplification of 16S rDNA, LacZ3, and ITS genes

**Fig. 3:**
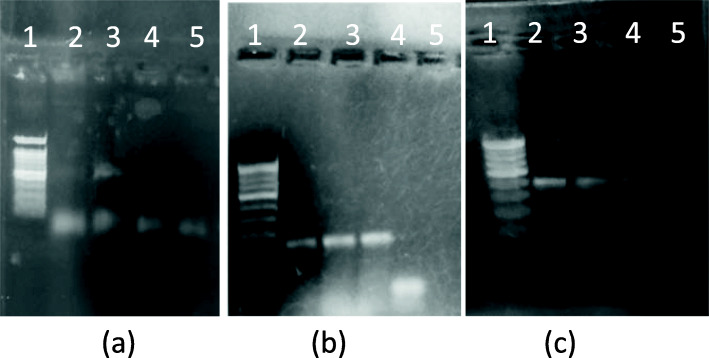
Annealing temperature optimization. **a** 16s r-DNA amplification (S. aureus CS): lane 1—100 bp ladder, lane 2—55 °C, lane 3—57 °C, lane 4—58 °C, lane 5—negative control. **b** β-galactocidase amplification using LacZ primers (E. coli. CS): lane 1—100 bp ladder, lane 2—55 °C, lane 3—57 °C, lane 4—58 °C, lane 5—negative control. **c** ITS amplification using ITS primers (C. albicans CS): lane 1—100bp ladder, lane 2—55 °C, lane 3—57 °C, lane 4—58 °C, lane 5—negative control. (L): 100 bp ladder

Annealing temperature for all three sets of primers was optimized by setting up a gradient PCR reaction with the temperatures 56, 57, and 58 °C. Colony suspension was spotted on the paper which served as a template DNA. As is seen in Fig. 3, good intensity amplicon was visible in PCR when the primer annealing temperature was 57 °C for amplification of all three genes. *S. aureus* colony suspension served as template for 16S rDNA detection whereas *E. coli* and *C. albicans* colony suspensions were used for detection of LacZ3 and ITS genes respectively.

### Paper size and culture volume optimization

**Fig. 4:**
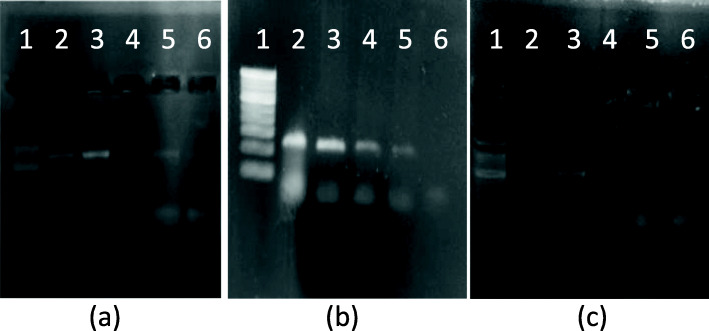
Paper size and culture volume optimization. **a** 16s r-DNA: lane 1—100 bp ladder, lane 2—colony PCR, lane 3—1 mm × 1 mm size ppr with 2 μL CS, lane 4—3 mm × 3 mm size ppr with 3 μL CS, lane 5—5 mm × 5 mm size ppr with 5 μL CS, lane 6—negative control. **b** β-galactocidase lane 1—100 bp ladder, lane 2—colony PCR, lane 3—1 mm × 1 mm size ppr with 2 μL CS, lane 4—3 mm × 3 mm size ppr with 3 μL CS, lane 5—5 mm × 5 mm size ppr with 5 μL CS, lane 6—negative control. **c** ITS lane 1—100 bp ladder, lane 2—colony PCR, lane 3—1 mm × 1 mm size ppr with 2 μL CS, lane 4—3 mm × 3 mm size ppr with 3 μL CS, lane 5—5 mm × 5 mm size ppr with 5 μL CS, lane 6—negative control

During the development of paper-based PCR method, it was important to keep the paper size to minimal to avoid the absorption of PCR master mix when the paper is dipped in the master mix. Also, it was critical to evaluate the lowest sample volume that can still detect specific gene. As depicted in Fig. 4, spotting of as low as 2 μL of colony suspension could detect all three genes. Hence, 2 μL culture spotted on 1 mm × 1 mm size Whatman paper no. 1 was used as template in all the future experiments.

### Master mix volume variation

**Fig. 5:**
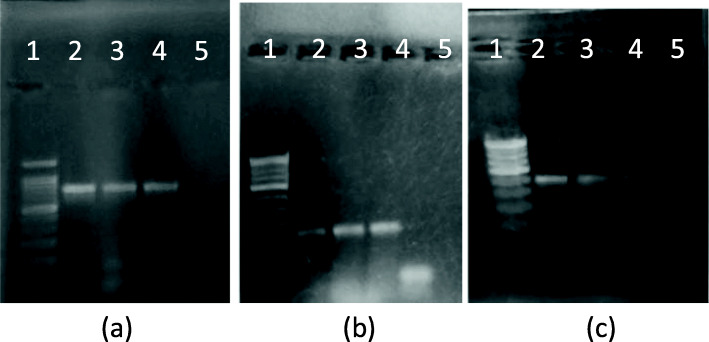
PCR master mix volume variation. **a** 16s r-DNA: lane 1—100 bp ladder, lane 2—colony PCR, lane 3—25 μL master mix volume, lane 4—50 μL master mix volume, lane 5—negative control. **b** β-galactocidase lane 1—100 bp ladder, lane 2—colony PCR, lane 3—25 μL master mix volume, lane 4—50 μL master mix volume, lane 5—negative control. **c** ITS: lane 1—100 bp ladder, lane 2—colony PCR, lane 3—25 μL master mix volume, lane 4—50 μL master mix volume, lane 5—negative control

Two independent reactions were set up having 25 μL and 50 μL master mix, and the same amount (2 μL) of colony suspension was spotted on filter paper and dipped in the master mix for the amplification of each gene. Both the PCR reactions gave strong amplicons (Fig. 5). Master mix volume did not impact the amplification. It was also evident that the same quantity of colony suspension could give result in large volume of master mix. Hence, master mix volume of 25 μL was used in future reactions.

### Specificity of primers used for 16s rDNA, LacZ3, and ITS regions

**Fig. 6:**
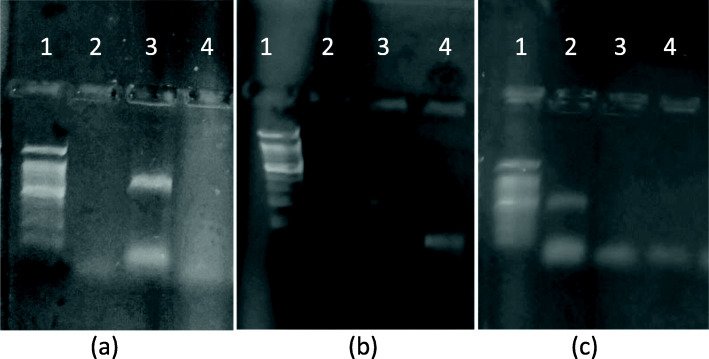
Specificity of primers. **a** 16s r-DNA: lane 1—100 bp ladder, lane 2—16s rDNA with C. albicans culture, lane 3—16s rDNA with S. aureus culture, lane 4—negative control. **b** β-galactocidase: lane 1—100 bp ladder, lane 2—β-gal with S. typhi, lane 3—β-gal with E. coli., lane 4—negative control. **c** ITS: lane 1—100 bp ladder, lane 2—ITS with C. albicans., lane 3—ITS with E. coli, lane 4—negative control

The selected primers were evaluated for their specificity to amplify respective gene region. As is seen in Fig. 6, all three sets of primers were highly specific as there was no non-specific amplification seen in any case.

### Application of developed PCR method for the detection of microbial contamination in milk samples

**Fig. 7:**
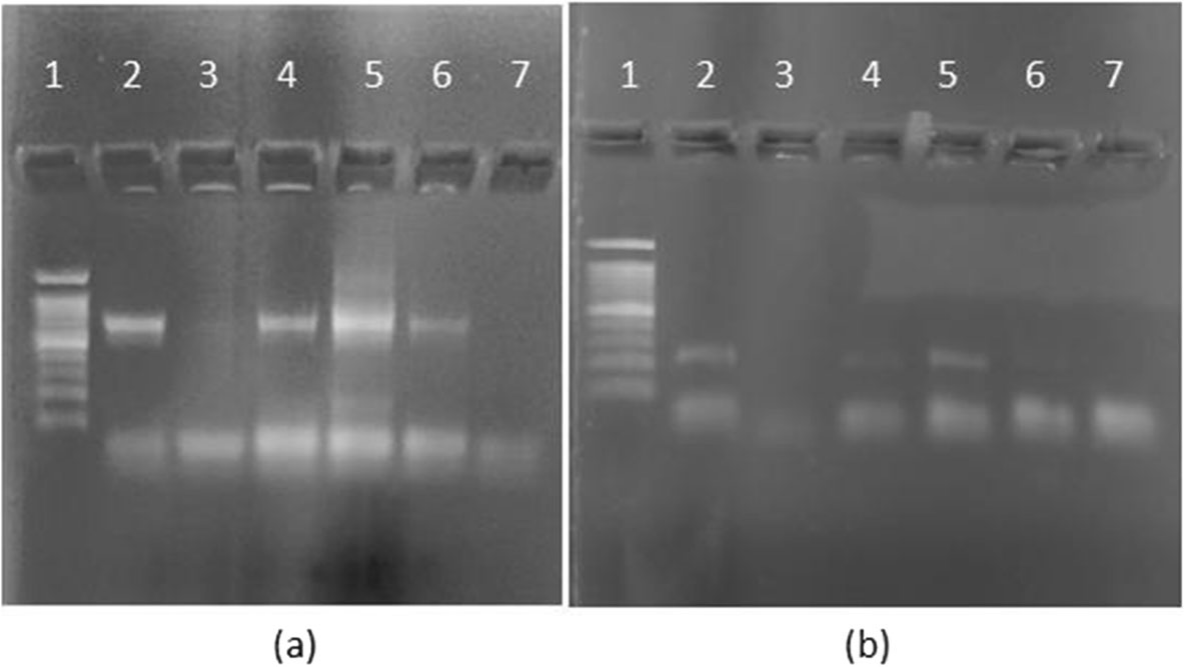
Detection of microbial contaminants in milk sample by PCR. **a**16s r-DNA: lane 1—100 bp ladder, lane 2—16s colony PCR (positive control), lane 3—Amul milk, lane 4—Gokul milk, lane 5—local cow milk, lane 6—local buffalo milk, lane 7—negative control. **b** β-galactocidase: lane 1—100 bp ladder, lane 2—β-galactocidase colony PCR (positive control), lane 3—Amul milk, lane 4—Gokul milk, lane 5—local cow milk, lane 6—local buffalo milk, lane 7—negative control

As mentioned in the materials and methods section, 2 μL of milk samples were directly spotted on the pre-sterilized Whatman filter paper square and allowed to dry. These squares were directly dipped in PCR master mix to detect bacterial contamination by amplifying 16S rDNA gene and coliform contamination by amplifying LacZ gene. As seen in Fig. 7, both the milk samples from local dairy could detect the presence of bacteria and coliforms as both the genes got amplified showing the respective amplicons on the gel. Similar results were obtained with milk from Gokul brand which was stored for 1 day. Interestingly, Amul milk did not show presence of coliforms as well as any other bacteria. These results perfectly matched with the data obtained through microbial analysis (CFU/mL) as mentioned in Table 1.

**Table 1:** Milk sample microbial contaminant CFU/mL count

Sr. no.	Milk sample	CFU/mL
1	Local cow milk	5.3 × 10^6^
2	Local buffalo milk	6.6 × 10^6^
3	Amul milk	Clear
4	Gokul milk	16 × 10^6^

## Discussion

Over the last 50 years, filter paper has gained an increasingly important role as a substrate for the diagnosis and surveillance of infectious diseases. Recently, this role has gone beyond diagnosis to include detection of markers of resistance, detailed genetic or serological analysis, and monitoring of therapeutic interventions, including drug levels, vaccine-induced responses, and viral loads. Point-of-care tests are increasingly providing a key role in diagnosing and surveying infectious diseases in remote settings, and affordable microfluidics devices based on paper to diagnose infectious diseases are becoming the promising tools [18]. The focus of the current research work was to develop a PCR method for the detection of microbes directly from the sample without the need to process the sample for isolation of genomic DNA. Whatman paper no. 1 was used as a carrier matrix for the sample. Hence, various parameters like primers, annealing temperature, and culture volume were optimized. Further, the developed method was also validated for its specificity and robustness.

For the detection of bacteria, a pair of universal primer was selected from the highly conserved region of the 16S rDNA sequence. These primers amplified the region which gave the amplicon of size of 709 bp. Earlier, these primers were also used by Matsuda et al. [17] for suspected blood infections and by Liu et al. [30] for the bacterial infection in CSF. Primers for 16S rDNA amplification were also used by Sauer et al. [25] for the detection of pathogenic bacteria causing prosthetic joint infections. The authors have claimed that the primer pair is highly specific for a broad range of bacterial pathogens and can be used with a variety of clinical specimens. The aim of this study was to establish the primer pair which is suitable for amplification of 16S rDNA gene which can be used commonly for the detection of broad range of bacteria. Similarly, for the detection of fungi and coliforms primers specific for ITS and LacZ region were selected. The ITS primers make use of conserved region of the 18 s, 5.8 s, and 28 s rRNA genes to amplify the noncoding regions between them [26]. All these primers could amplify respective genes when colony suspension was used as a source of template. This step was crucial as it could avoid the sample processing step which can delay the detection as well as the processing step can impact the DNA quality. Hence, the colony suspension was used in subsequent paper-based method as template source. During the method development, the colony suspension served as a representation of sample source. Colony PCR is a very well established technique and in the current study, adding colony suspension to PCR master mix has also given expected results. While developing the PCR method where Whatman paper would serve as a carrier matrix for sample, authentic source of bacterial culture was required as a template to optimize the amplification of the selected genes. Hence, the colony suspension was spotted on the paper directly and the optimization of various parameters of PCR was done using this as a template.

For any PCR reaction, primer annealing temperature plays a critical role in ensuring the optimal amplification as well as avoiding non-specific binding. Hence, for the developed PCR reaction, it was important to optimize the annealing temperature that is suitable for all three sets of primers. The recommended annealing temperatures for 16S rDNA universal primers used in this study was 55 °C, for LacZ3 was 57 °C, and the ITS gene was 55 °C. To optimize this annealing temperature for better amplification, a gradient PCR was set up with the range 56, 57, and 58 °C. When the colony suspensions spotted on the Whatman paper no. 1 were used as a template source in PCR, good intensity amplicon was visible when the primer annealing temperature was 57 °C for all three genes. Standard colony PCR without the use of Whatman filter paper served as a positive control.

Another critical parameter that was evaluated during the development of this paper-based PCR method was the volume of culture spotted on the paper. This is important because the lowest volume of the culture required to give amplification of the gene will also reduce the size of the paper used for spotting. Larger size paper poses the problem of absorption of master mix from the tube which hampers the results of the reaction. Earlier studies by Lokur et al. [31] have also shown culture volume optimization in the paper-based PCR method for amplification of 16srDNA gene. However, the authors have not evaluated the paper size, which was unique to this study. In the current study, the paper dimension and the volume of culture were varied and the PCR reaction was set up. The amplification of 16S rDNA, LacZ3, and ITS genes could be achieved with 2 μL culture spotted on 1 mm × 1 mm size paper. Additionally, PCR master mix volume optimization studies showed that master mix volume did not impact the amplification.

As per ICH guidelines, it is crucial to establish specificity of the newly developed method for the desired end result [32]. The specificity of 16S rDNA, LacZ3, and ITS primers for the detection of bacteria, coliforms, and fungi was established. The efficiency of the developed paper-based PCR method was tested by using the method for the detection of contaminating microbes in different milk samples. These milk samples were also tested using traditional microbiological methods for the detection of contaminants. There are several methods available for counting viable bacterial cells in milk [33]. Different ISO methods are also available to quantify probiotics and fermenting microbes employed in the dairy industry [34].

In the current study, we used plate count method. Viable bacterial cell count in all the dilutions of milk sample was determined by the method described by Miles and Mishra [29]. The CFU/mL was calculated as the indicator of microbial presence in case of each milk sample (Table 1). As is evident from the data mentioned in Table 1, cow and buffalo milk samples from the local diary showed significant microbial load. Additionally, milk from Gokul brand also showed presence of microbes probably because the milk was purposely stored for a day till it starts spoiling. Whereas pasteurized and cold-stored Amul milk did not show any microbial presence. None of the milk samples showed any fungal growth on Sabouraud Dextrose (SD) agar even after 48 h of incubation. However, both the types of milk from local dairy and Gokul milk showed pink colonies on MacConkey’s agar which is indicative of coliform presence. It was important to compare the above results that were obtained through known microbial methods with the developed paper-based PCR method. This was important as it would validate the developed method for the detection of microbes. Most importantly, in paper-based PCR method, there was no sample preparation required neither DNA extraction step was necessary to get the results. Important finding of this study was none of the milk protein and fat interfered with the PCR reaction although the milk was directly spotted without any prior treatment. Also, the PCR method could detect both bacterial and coliform contamination in the samples with a really small volume of 2 uL which had approximately 10^4^ cells. Microbial methods would not be able to pick the contaminants at such a low cell number. Fu et al. [35] have also shown that a paper-based chip device could detect pathogens like *Listeria* very efficiently when CFU count was as low as 10^4^ cells/mL. The microbial results usually need 24–48 h of incubation whereas the PCR takes about 2 h to give the same data. The data thus confirms that developed PCR method is absolutely comparable to the traditional microbial methods and is faster, less time consuming, and highly sensitive.

## Conclusion

In summary, we have developed a novel paper-based PCR method for the detection of coliforms, bacterial, and fungal contamination in food samples. The method is easy to handle with no sample preparation requirement. The testing time is less about 2 to 3 h, and the method can detect as low as 200 cells. Also, the use of positive and negative controls in all our experiments excluded the possibility of false-positive and false negatives, which may lead to misinterpretation of the results.

The most promising part of the method is that no sample preparation is required and sample components are not interfering with the PCR reaction. Of course, it needs to be seen if samples with turbidity, strong color, or different pH would behave the same way. In that case, it would be very easy to collect the samples from a remote area and can be brought to testing facility with no specific transportation conditions or temperature requirements. A non-technical person also can collect the samples. The sample carrier Whatman paper no. 1 can also be disposed easily after completion of the test. The current method platform can be adapted and integrated with further developments in the detection of other bacteria and pathogens, and used not just for milk samples but many other products like potable water, wine, and juices.

## Data Availability

All the data generated and/or analyzed during this study are included in this published article.

## Abbreviations

PCR: Polymerase chain reaction
LB: Luria–Bertani
MA: MacConkey agar
PDA: Potato dextrose agar
LAFU: Laminar air flow unit
g.DNA: Genomic DNA
β-gal: Beta galactocidase
ITS: Internal transcribed spacer
